# Whole-Genome Sequence of *Bradyrhizobium elkanii* Strain UASWS1016, a Potential Symbiotic Biofertilizer for Agriculture

**DOI:** 10.1128/genomeA.01095-16

**Published:** 2016-10-06

**Authors:** Julien Crovadore, Gautier Calmin, Romain Chablais, Bastien Cochard, Torsten Schulz, François Lefort

**Affiliations:** a Plants and Pathogens Group, Research Institute Land Nature and Environment, Hepia, HES-SO University of Applied Sciences and Arts Western Switzerland, Geneva, Switzerland; b Faculty of Engineering and Architecture, HES-SO University of Applied Sciences and Arts Western Switzerland, Delémont, Switzerland; c Philotimo S.A., Geneva, Switzerland

## Abstract

*Bradyrhizobium elkanii* UASWS1016 has been isolated from a wet oxidation sewage plant in Italy. Fully equipped for ammonia assimilation, heavy metal resistances, and aromatic compounds degradation, it carries a large type IV secretion system, specific of plant-associated microbes. Deprived of toxins, it could be considered for agricultural and environmental uses.

## GENOME ANNOUNCEMENT

The bacterium *Bradyrhizobium elkanii*, described in 1992 (1), is a symbiotic organism which forms root nodules in various hosts. These bacteria are aerobic, motile, Gram-negative rods, which do not form spores and are found as free-living organisms or plant symbionts. Used for producing bioemulsifiers (2), they are mainly applied as an inoculated or natural biofertilizer in symbiosis with important legume crops, such as soybean, cowpea, mung bean, and acacia (3–5). The strain UASWS1016 has been isolated from the sediment of a wet oxidation installation through selection for highly ammonia-tolerant nitrifying bacteria. Identified as *Bradyrhizobium elkanii* by 16S rDNA sequencing, it shares 100% identity for this gene with 30 *Bradyrhizobium elkanii* strains registered in GenBank (6). Genomic DNA was extracted from a pure axenic culture following an adapted protocol (7). Libraries were created using the TruSeq DNA PCR-free library preparation kits (Illumina, USA). Whole-genome shotgun (WGS) sequencing was carried out within one Illumina MiniSeq run at 2 × 150 bp paired-end read length, using a MiniSeq mid output kit (300 cycles) which provided a 92× genome coverage. Quality control of the reads was assessed with FastQC (8). Genome assembly was computed with SPAdes Genome assembler 3.8.1 (9) and produced 59 contigs (≥200 bp). These contigs were arranged with BioEdit (10) and analyzed with QUAST (11). The total length of the genome was 7,960,052 bp, with a G+C content of 64.62% and an *N*_50_ value of 386,652 bp. PlasmidSPAdes (12) detected three plasmids of 7,997 bp, 26,868 bp, and 58,330 bp, in length, respectively. Automated gene annotation, carried out by the NCBI Prokaryotic Genome Automatic Annotation Pipeline PGAAP (13), allowed for the identification of 7,376 genes distributed in 7,309 coding sequences (CDS), 76 pseudogenes, and 67 RNA genes. Annotation with RAST 2.0 (14, 15) identified 7,538 CDS spread over 499 subsystems. No transposons or phages were found. Since genes of toxins, superantigens, virulence, and disease are absent, this bacterium could be considered a potential fertilizing agent. *B. elkanii* strains harbor type III and type IV secretion systems (16), but the strain UASWS1016, like the strain UASWS1015 (17), only displayed a large type IV secretion system, specific of plant-microbes associations, composed of 31 genes for Vir proteins (18). Additionally, it is equipped with 10 genes for bacteriocin and antimicrobial synthesis as well as 145 genes involved in antibiotics and multidrug and heavy metal resistances. The bacterium is fully equipped for ammonia assimilation. Additionally, 147 genes are involved in degradation of aromatic compounds, which offers the possible capacity to grow in polluted soils. The presence of genes involved in plant auxins synthesis (five genes), inorganic and organic sulfur assimilation (91 genes), phosphorus metabolism (86 genes), and organic acids (20 genes) should provide desired characteristics of plant growth-promoting rhizobacteria (PGPR) (19). A few genes of nodulation (nodD, nodN, noIO, nodT) are present but the most important nod genes A, B, and C (20) are absent. *Bradyrhizobium* strains without nod genes are, however, able to nodulate (21). This genome will add to the knowledge of this important species for agriculture.

### Accession number(s).

This WGS project was deposited at DDBJ/EMBL/GenBank under the accession number MDEP00000000. The version described in this paper is the first version, MDEP00000000.1. The 59 contigs have been deposited under the accession numbers MDEP01000001 to MDEP01000059.
